# Synthesis and Thermal Characteristic of Liquid Crystalline Polyoxetane Containing Trans-Stilbene Side Group

**DOI:** 10.3390/polym12010185

**Published:** 2020-01-10

**Authors:** Li-Chuan Wu, Cheng-Chih Chen, Chih-Hung Lin

**Affiliations:** 1 Department of Applied Chemistry and Material Sciences, Fooyin University, 151 Jinxue Road, Daliao, Kaohsiung City 83102, Taiwan; SC023@fy.edu.tw; 2 Center for General Education, Chang Gung University of Science and Technology, 261 Wen-Hwa 1st Road, Kwei-Shan, Tao-Yuan 33303, Taiwan; ccchen@gw.cgust.edu.tw; 3 Research Center for Food and Cosmetic Safety and Research Center for Chinese Herbal Medicine, Chang Gung University of Science and Technology, Kweishan, Tao-Yuan 33303, Taiwan

**Keywords:** polyoxetane, liquid crystal, stilbene

## Abstract

A series of fourteen liquid crystalline monomers and polyoxetanes containing trans-biphenyl side group have been successfully synthesized. The thermal and mesomorphic properties of monomers (**1M**~**14M**) and polymers (**1P**~**14P**) are measured using DSC, POM, and X-ray. All of the series monomers present enantiotropic smectic H and smectic G phase and the series polymers show enantiotropic smectic A phase which three polymers contained exhibit smectic E. Polyoxetanes have been used as a cationic ring-opening polymerization of oxetane monomers bearing a pendant trans-stilbene mesogenic unit including different spacer length and terminal alkyl length.

## 1. Introduction

The first thermotropic side-chain liquid crystalline polymers were synthesized by Finkelmann and Rehage [1,2]. They had already understood the main factors in the formation of the liquid crystal phase of compounds. The liquid crystal properties of the compound were affected by the backbone liquid crystal structure, the mesogenic unit, the tail group, and the spacer length. The side-chain liquid crystal polymer integrated the properties of the liquid crystal and the polymer properties. They had potential applications in optical data storage, piezoelectric transducer, nonlinear optics, and gas or liquid chromatography stationary phases [3,4,5,6,7].

In the past few decades, a large number of side-chain liquid crystal polymers had been synthesized [8,9,10]. They combined the many different backbone types (such as methacrylates, acrylates, siloxanes, epoxides, ethylenes, etc.) and the vast number of mesogenic units available. Kawakami et al. reported the first example of a cationic ring-opening polymerized side-chain liquid crystalline polyoxetane [11,12,13,14,15,16].

According to the experimental results, polyoxetane flexibility had more than polyacrylate and polymethacrylate. The polymerization of the side-chain liquid crystalline polyoxetane molecular weight distribution (MWD) is less than 1.3.

The purpose of this research is to show the synthesis of a new series of side-chain liquid crystalline polyoxetanes containing the trans-stilbene mesogenic side group. The effects of terminal alkyl length and spacer length on the properties of mesophases exhibited are discussed.

## 2. Materials and Methods

### 2.1. Instruments

^1^H-NMR (400 MHz) spectra were measured using a Bruker AM 400 instrument (Bruker, Daltonik, Germany). The thermal transitions and the anisotropic textures were measured using a Carl-Ziess Axiphot polarized optical microscope (Carl-Ziess, Jena, Germany) and a Mettler FP82 hot stage (Mettler, Switzerland). Differential scanning calorimeter (DSC) was recorded on a Seiko SSC/5200 (Seiko, New Castle, DE, USA) with determined compounds of thermal transitions and thermodynamic parameters equipped with a cooling accessory. Thermal stability was measured using a Seiko TG/DTA 200 thermal gravimetric analyzer (Seiko, New Castle, DE, USA). X-ray diffraction by liquid crystals was measured using a Riraku powder diffractometer (Riraku, Austin, TX, USA).

### 2.2. Synthesis

**Scheme 1:**
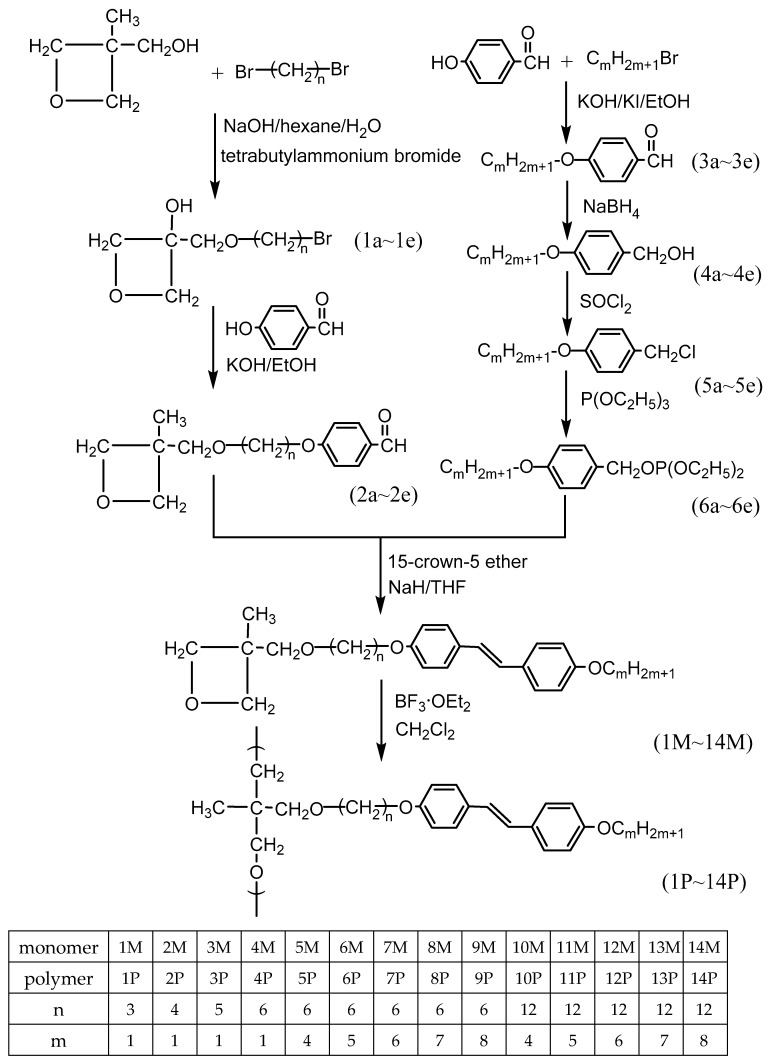
Synthesis of monomers **1M**~**14M** and polymers **1P**~**14P**.

The intermediates and targets compound synthetic routes were represented in Scheme 1. TLC and ^1^H-NMR spectroscopy were verified as the chemical structures and purity of the intermediates and target compounds. The synthesis methods and analysis of each product are described below.

3-[(3-Bromopropoxy)methyl]-3-methyloxetane (**1a**)

3-[(4-Bromobutoxy)methyl]-3-methyloxetane (**1b**)

3-[(5-Bromopentoxy)methyl]-3-methyloxetane (**1c**)

3-[(6-Bromohexoxy)methyl]-3-methyloxetane (**1d**)

3-[(12-Bromododecoxy)methyl]-3-methyloxetane (**1e**)

All five compounds were prepared by the same method. Taking compound **1d** as an example, the synthesis is described below.

3-(Hydroxymethyl)-3-methyloxetane (10.0 g, 0.098 mol), dibromohexane (73.2 g, 0.299 mol), and hexane (120 mL) was added to a stirred solution of sodium hydroxide (64.7 g, 1.618 mol) in 150 mL of water. Then, tetrabutylammonium bromide (1.0 g) was added to the solution. The solution was stirred for 12 h at room temperature, then heated to reflux for 0.5 h. The reaction solution cooled to room temperature, 1000 mL of water was added, and the organic layer was extracted three times with hexane. The extraction solution was dried through anhydrous magnesium sulfate and after removal of the solvent under reduced pressure. The crude product was purified by distillation, to yield 20.85 g (80.3%) of a colorless transparent liquid. ^1^H-NMR (300 MHz, CDCl_3_, δ, ppm): 1.30 (s, 3H, –CH_3_ on the oxetane ring), 1.46–1.90 (m, 8H, –OCH_2_(CH_2_)_4_CH_2_–), 3.39–3.48 (m, 6H, –CH_2_OCH_2_(CH_2_)_4_CH_2_Br), 4.34, 4.51 (AB quartet, each 2H, –CH_2_–O on the oxetane ring).

3-[[3-(4-Hydroxybenzaldehyde)propoxy]methyl]-3-methyl oxetane (**2a**)

3-[[4-(4-Hydroxybenzaldehyde)butoxy]methyl]-3-methyl oxetane (**2b**)

3-[[5-(4-Hydroxybenzaldehyde)pentoxy]methyl]-3-methyl oxetane (**2c**)

3-[[6-(4-Hydroxybenzaldehyde)hexoxy]methyl]-3-methyl oxetane (**2d**)

3-[[12-(4-Hydroxybenzaldehyde)dodecanoxy]methyl]-3-methyl oxetane (**2e**)

4-Butoxy-benzaldehyde (**3a**)

4-Pentoxy-benzaldehyde (**3b**)

4-Hexoxy-benzaldehyde (**3c**)

4-Heptoxy-benzaldehyde (**3d**)

4-Octoxy-benzaldehyde (**3e**)

All ten compounds were prepared by the same method. Taking compound **2d** as an example, the synthesis is described below.

4-Hydroxybenzaldehyde (4.45 g, 0.036 mol) was added to a stirred solution of potassium hydroxide (2.04 g, 0.036 mol) and potassium iodide (0.2 g) in 100 mL of 95% ethanol. Compound **1d** (8.00 g, 0.030 mol) was added dropwise after the solution mentioned above was refluxed for 1 h. The solution refluxed for 12 h and then cooled to room temperature. The solution was extracted with water and ethyl acetate. The extraction solution was washed with 10% KOH solution three times and dried through anhydrous magnesium sulfate. After removal of the solvent under reduced pressure, the crude product was purified by column chromatography on silica gel using ethyl acetate/hexane as eluent to yield 2.42 g (82.5%) of light yellow liquid. ^1^H-NMR (300 MHz, CDCl_3_, δ, ppm): 1.30 (s, 3H, –CH_3_ on the oxetane ring), 1.40–1.82 (m, 8H, –OCH_2_(CH_2_)_4_CH_2_–), 3.46 (m, 4H, –CH_2_O–), 4.04 (t, 2H, –CH_2_–OPh), 4.33, 4.49 (AB quartet, each 2H, –CH_2_–O on the oxetane ring), 6.96 (d, 2H, aromatic protons), 7.81 (d, 2H, aromatic protons), 9.87 (s, 1H, aldehyde protons).

4-Butoxy-benzyl alcohol (**4a**)

4-Pentoxy-benzyl alcohol (**4b**)

4-Hexoxy-benzyl alcohol (**4c**)

4-Heptoxy-benzyl alcohol (**4d**)

4-Octoxy-benzyl alcohol (**4e**)

Taking compound **4c** as an example, the synthesis is described below.

A solution of sodium tetrahydridoborate (3.28 g, 0.087 mol) in 2 mL of 0.45 N sodium hydroxide with 28 mL water was slowly added dropwise to 4-Hexoxy-benzaldehyde (14.9 g, 0.072 mol) with methanol (150 mL) solution. The solution was stirred 3 h at room temperature. Remove most of the methanol by distillation. The solution was extracted with ether and aqueous of dilute acid solution (50 mL). The organic phase was washed with 2% aqueous of sodium bicarbonate, saturated aqueous of sodium chloride, and dried through anhydrous magnesium sulfate. The crude product was purified by column chromatography on silica gel using n-hexane/ethyl acetate as eluent to yield 13.8 g (92.1%) of colorless liquid. ^1^H-NMR (300 MHz, CDCl_3_, δ, ppm): 0.89 (t, 3H, –CH_3_), 1.31–1.80 (m, 8H, –OCH_2_(CH_2_)_4_CH_3_), 3.96 (t, 2H, –OCH_2_(CH_2_)_4_–), 4.66 (s, 2H, Ph–CH_2_Cl), 6.89 (d, 2H, aromatic protons), 7.30 (d, 2H, aromatic protons).

4-Butoxy-benzyl chloride (**5a**)

4-Pentoxy-benzyl chloride (**5b**)

4-Hexoxy-benzyl chloride (**5c**)

4-Heptoxy-benzyl chloride (**5d**)

4-Octoxy-benzyl chloride (**5e**)

All five compounds were prepared by the same method. Taking compound **5c** as an example, the synthesis is described below.

4-Hexoxy-benzyl alcohol (5 g, 0.024 mol) was reacted with excess thionyl chloride (15 mL, 0.0206 mol) in 50 mL of methylene chloride. The solution was stirred at ice bath for 6 h. Then, added with water (30 mL) into the solution. The organic phase was washed with 10% aqueous of sodium bicarbonate, saturated aqueous of sodium chloride, and dried through anhydrous magnesium sulfate. The crude product was purified by column chromatography on silica gel using n-hexane/ethyl acetate as eluent to yield 4.58 g (84.3%) of light-yellow liquid. ^1^H-NMR (300 MHz, CDCl_3_, δ, ppm): 0.89 (t, 3H, –CH_3_), 1.31–1.80 (m, 8H, –OCH_2_(CH_2_)_4_CH_3_), 3.96 (t, 2H, –OCH_2_(CH_2_)_4_–), 4.66 (s, 2H, Ph–CH_2_Cl), 6.89 (d, 2H, aromatic protons), 7.30 (d, 2H, aromatic protons).

Diethyl[(4-butoxy)benzyl]phosphonate (**6a**)

Diethyl[(4-pentoxy)benzyl]phosphonate (**6b**)

Diethyl[(4-hexoxy)benzyl]phosphonate (**6c**)

Diethyl[(4-heptoxy)benzyl]phosphonate (**6d**)

Diethyl[(4-octoxy)benzyl]phosphonate (**6e**)

All five compounds were prepared by the same method. Taking compound **6c** as an example, the synthesis is described below.

4-Hexoxy-benzyl chloride (5.00 g, 0.022 mol) was injected by triethyl phosphite (11.0 g, 0.066 mol) with a syringe under nitrogen. The reaction mixture was refluxed for 12 h, then, was purified by distillation under reduced pressure to yield 5.89 g (81.6%) of colorless liquid. ^1^H-NMR (300 MHz, CDCl_3_, δ, ppm): 0.89 (t, 3H, –CH_3_), 1.22 (m, 6H, P(OCH_2_CH_3_)_2_, 1.31–1.79 (m, 8H, –OCH_2_(CH_2_)_4_CH_3_), 3.05 (d, 2H, PCH_2_Ph), 3.93–4.01 (m, 6H, –OCH_2_(CH_2_)_4_– and P(OCH_2_CH_3_)_2_), 6.89 (d, 2H, aromatic protons), 7.30 (d, 2H, aromatic protons).

3-[3-(Trans-4′-methoxystilben-4-yloxy)propoxymethyl]-3-methyl oxetane (**1M**)

3-[4-(Trans-4′-methoxystilben-4-yloxy)butoxymethyl]-3-methyl oxetane (**2M**)

3-[5-(Trans-4′-methoxystilben-4-yloxy)pentoxymethyl]-3-methyl oxetane (**3M**)

3-[6-(Trans-4′-methoxystilben-4-yloxy)hexoxymethyl]-3-methyl oxetane (**4M**)

3-[6-(Trans-4′-butoxystilben-4-yloxy)hexoxymethyl]-3-methyl oxetane (**5M**)

3-[6-(Trans-4′-pentoxystilben-4-yloxy)hexoxymethyl]-3-methyl oxetane (**6M**)

3-[6-(Trans-4′-hexoxystilben-4-yloxy)hexoxymethyl]-3-methyl oxetane (**7M**)

3-[6-(Trans-4′-heptoxystilben-4-yloxy)hexoxymethyl]-3-methyl oxetane (**8M**)

3-[6-(Trans-4′-octoxystilben-4-yloxy)hexoxymethyl]-3-methyl oxetane (**9M**)

3-[12-(Trans-4′-butoxystilben-4-yloxy)dodecoxymethyl]-3-methyl oxetane (**10M**)

3-[12-(Trans-4′-pentoxystilben-4-yloxy)dodecoxymethyl]-3-methyl oxetane (**11M**)

3-[12-(Trans-4′-hexoxystilben-4-yloxy)dodecoxymethyl]-3-methyl oxetane (**12M**)

3-[12-(Trans-4′-heptoxystilben-4-yloxy)dodecoxymethyl]-3-methyl oxetane (**13M**)

3-[12-(Trans-4′-octoxystilben-4-yloxy)dodecoxymethyl]-3-methyl oxetane (**14M**)

All fourteen monomers **1M**~**14M** were prepared by the same method. Taking monomer **7M** as an example, the synthesis is described below.

Sodium hydride (0.33 g, 0.013 mol) dissolved in dry THF (50 mL) on the brown flask, 13-crown-5-ether (30 mg) was added to react under nitrogen in the ice bath. Then, a solution of compound **2d** (2.52 g, 0.008 mol) and compound **6d** (2.6 g, 0.008 mol) was added dropwise to a stirred mixture. The reaction mixture was stirred for 12 h at room temperature. The reaction solution mixture was poured into ice water. The solution was filtered, the remaining yellow solid was recrystallized from dimethyl formamide to yield 1.62 g of light-yellow solid.

The ^l^H-NMR spectrometer, the product yield, and element analysis of monomers **1M**~**14M** were as follows.

Compound **1M**: Yield: 40.7%; ^1^H-NMR (300 MHz, CDCl_3_, δ, ppm): 1.28 (s, 3H, –CH_3_ on the oxetane ring), 2.03 (t, 2H, –OCH_2_CH_2_–), 3.48 (s, 2H, –CH_2_O(CH_2_)_3_–), 3.62 (t, 2H, –CH_2_OCH_2_–), 3.80 (s, 3H, –PhOCH_3_), 4.04 (t, 2H, –CH_2_CH_2_OPh), 4.32, 4.48 (AB quartet, each 2H, –CH_2_– on the oxetane ring), 6.84, 7.38 (m, 6H, 4H, stilbene protons); element analysis: Calc. for C_23_H_28_O_4_: C 75.00, H 7.61, O 17.39; found C 75.00, H 7.75, O 17.25%.

Compound **2M**: Yield: 38.6%; ^1^H-NMR (300 MHz, CDCl_3_, δ, ppm): 1.31 (s, 3H, –CH_3_ on the oxetane ring), 1.79 (m, 4H, –OCH_2_(CH_2_)_2_–), 3.49 (t, 4H, –CH_2_OCH_2_–), 3.82 (s, 3H, –PhOCH_3_), 3.98 (t, 2H, –CH_2_CH_2_OPh), 4.34, 4.50 (AB quartet, each 2H, –CH_2_– on the oxetane ring), 6.85, 7.39 (m, 6H, 4H, stilbene protons); element analysis: Calc. for C_24_H_30_O_4_: C 75.39, H 7.85, O 16.75; found C 75.46, H 7.99, O 16.45%.

Compound **3M**: Yield: 33.4%; ^1^H-NMR (300 MHz, CDCl_3_, δ, ppm): 1.34 (s, 3H, –CH_3_ on the oxetane ring), 1.58–1.87 (m, 6H, –OCH_2_(CH_2_)_3_–), 3.51 (t, 4H, –CH_2_OCH_2_–), 3.85 (s, 3H, –PhOCH_3_), 3.99 (t, 2H, –CH_2_CH_2_OPh), 4.38, 4.53 (AB quartet, each 2H, –CH_2_– on the oxetane ring), 6.85, 7.43 (m, 6H, 4H, stilbene protons); element analysis: Calc. for C_25_H_32_O_4_: C 75.76, H 8.08, O 16.16; found C 75.52, H 8.15, O 16.33%.

Compound **4M**: Yield: 30.8%; ^1^H-NMR (300 MHz, CDCl_3_, δ, ppm): 1.31 (s, 3H, –CH_3_ on the oxetane ring), 1.47–1.85 (m, 8H, –OCH_2_(CH_2_)_4_–), 3.46 (t, 4H, –CH_2_OCH_2_–), 3.81 (s, 3H, –PhOCH_3_), 3.99 (t, 2H, –CH_2_CH_2_OPh), 4.34, 4.50 (AB quartet, each 2H, –CH_2_– on the oxetane ring), 6.87, 7.26 (m, 6H, 4H, stilbene protons); element analysis: Calc. for C_26_H_34_O_4_: C 76.10, H 8.29, O 15.61; found C 75.82, H 8.47, O 15.71%.

Compound **5M**: Yield: 40.6%; ^1^H-NMR (300 MHz, CDCl_3_, δ, ppm): 0.96 (t, 3H, –CH_2_–CH_3_), 1.31 (s, 3H, –CH_3_ on the oxetane ring), 1.47–1.80 (m, 12H, –OCH_2_(CH_2_)_2_CH_3_; –OCH_2_(CH_2_)_4_CH_2_–), 3.46 (t, 4H, –CH_2_OCH_2_–), 3.94 (t, 4H, –CH_2_–OPh–), 4.35, 4.51 (AB quartet, each 2H, –CH_2_– on the oxetane ring), 6.86, 7.39 (m, 6H, 4H, stilbene protons); element analysis: Calc. for C_29_H_40_O_4_: C 76.99, H 8.85, O 14.16; found C 77.03, H 9.02, O 13.95%.

Compound **6M**: Yield: 36.7%; ^1^H-NMR (300 MHz, CDCl_3_, δ, ppm): 0.91 (t, 3H, –CH_2_–CH_3_), 1.31 (s, 3H, –CH_3_ on the oxetane ring), 1.41–1.79 (m, 14H, –OCH_2_(CH_2_)_3_CH_3_; –OCH_2_(CH_2_)_4_CH_2_–), 3.45 (t, 4H, –CH_2_OCH_2_–), 3.94 (t, 4H, –CH_2_–OPh–), 4.35, 4.50 (AB quartet, each 2H, –CH_2_– on the oxetane ring), 6.86, 7.39 (m, 6H, 4H, stilbene protons); element analysis: Calc. for C_30_H_42_O_4_: C 77.25, H 9.01, O 13.73; found C 77.21, H 9.15, O 13.64%.

Compound **7M**: Yield: 41.0%; ^1^H-NMR (300 MHz, CDCl_3_, δ, ppm): 0.91 (t, 3H, –CH_2_–CH_3_), 1.31–1.80 (m, 19H, –CH_3_ on the oxetane ring, –OCH_2_(CH_2_)_4_CH_3_, –OCH_2_(CH_2_)_4_CH_2_–), 3.46 (t, 4H, –CH_2_OCH_2_–), 3.94 (t, 4H, –CH_2_–OPh–), 4.35, 4.50 (AB quartet, each 2H, –CH_2_– on the oxetane ring), 6.86, 7.39 (m, 6H, 4H, stilbene protons); element analysis: Calc. for C_31_H_44_O_4_: C 77.50, H 9.17, O 13.33; found C 77.47, H 9.26, O 13.27%.

Compound **8M**: Yield: 40.6%; ^1^H-NMR (300 MHz, CDCl_3_, δ, ppm): 0.88 (t, 3H, –CH_2_–CH_3_), 1.26–1.80 (m, 21H, –CH_3_ on the oxetane ring, –OCH_2_(CH_2_)_5_CH_3_, –OCH_2_(CH_2_)_4_CH_2_–), 3.46 (t, 4H, –CH_2_OCH_2_–), 3.95 (t, 4H, –CH_2_–OPh–), 4.35, 4.50 (AB quartet, each 2H, –CH_2_– on the oxetane ring), 6.86, 7.39 (m, 6H, 4H, stilbene protons); element analysis: Calc. for C_32_H_46_O_4_: C 77.73, H 9.31, O 12.96; found C 77.60, H 9.40, O 13.00%.

Compound **9M**: Yield: 38.6%; ^1^H-NMR (300 MHz, CDCl_3_, δ, ppm): 0.87 (t, 3H, –CH_2_–CH_3_), 1.26–1.82 (m, 23H, –CH_3_ on the oxetane ring, –OCH_2_(CH_2_)_6_CH_3_, –OCH_2_(CH_2_)_4_CH_2_–), 3.46 (t, 4H, –CH_2_OCH_2_–), 3.94 (t, 4H, –CH_2_–OPh–), 4.35, 4.50 (AB quartet, each 2H, –CH_2_– on the oxetane ring), 6.86, 7.39 (m, 6H, 4H, stilbene protons); element analysis: Calc. for C_33_H_48_O_4_: C 77.95, H 9.45, O 12.60; found C 77.92, H 9.54, O 12.54%.

Compound **10M**: Yield: 34.7%; ^1^H-NMR (300 MHz, CDCl_3_, δ, ppm): 0.96 (t, 3H, –CH_2_–CH_3_), 1.29–1.79 (m, 27H, –CH_3_ on the oxetane ring, –OCH_2_(CH_2_)_2_CH_3_, –OCH_2_(CH_2_)_10_CH_2_–), 3.43 (t, 4H, –CH_2_OCH_2_–), 3.96 (t, 4H, –CH_2_–OPh–), 4.35, 4.50 (AB quartet, each 2H, –CH_2_– on the oxetane ring), 6.86, 7.39 (m, 6H, 4H, stilbene protons); element analysis: Calc. for C_35_H_52_O_4_: C 78.36, H 9.70, O 11.94; found C 78.22, H 9.80, O 11.98%.

Compound **11M**: Yield: 39.1%; ^1^H-NMR (300 MHz, CDCl_3_, δ, ppm): 0.91 (t, 3H, –CH_2_–CH_3_), 1.27–1.78 (m, 29H, –CH_3_ on the oxetane ring, –OCH_2_(CH_2_)_3_CH_3_, –OCH_2_(CH_2_)_10_CH_2_–), 3.43 (t, 4H, –CH_2_OCH_2_–), 3.94 (t, 4H, –CH_2_–OPh–), 4.34, 4.50 (AB quartet, each 2H, –CH_2_– on the oxetane ring), 6.86, 7.39 (m, 6H, 4H, stilbene protons); element analysis: Calc. for C_36_H_54_O_4_: C 78.54, H 9.82, O 11.64; found C 78.26, H 9.90, O 11.84%.

Compound **12M**: Yield: 24.1%; ^1^H-NMR (300 MHz, CDCl_3_, δ, ppm): 0.90 (t, 3H, –CH_2_–CH_3_), 1.26–1.79 (m, 31H, –CH_3_ on the oxetane ring, –OCH_2_(CH_2_)_4_CH_3_, –OCH_2_(CH_2_)_10_CH_2_–), 3.43 (t, 4H, –CH_2_OCH_2_–), 3.94 (t, 4H, –CH_2_–OPh–), 4.34, 4.50 (AB quartet, each 2H, –CH_2_– on the oxetane ring), 6.86, 7.39 (m, 6H, 4H, stilbene protons); element analysis: Calc. for C_37_H_56_O_4_: C 78.72, H 9.93, O 11.35; found C 78.40, H 10.05, O 11.55%.

Compound **13M**: Yield: 27.4%; ^1^H-NMR (300 MHz, CDCl_3_, δ, ppm): 0.87 (t, 3H, –CH_2_–CH_3_), 1.26–1.77 (m, 33H, –CH_3_ on the oxetane ring, –OCH_2_(CH_2_)_5_CH_3_, –OCH_2_(CH_2_)_10_CH_2_–), 3.44 (t, 4H, –CH_2_OCH_2_–), 3.95 (t, 4H, –CH_2_–OPh–), 4.35, 4.49 (AB quartet, each 2H, –CH_2_– on the oxetane ring), 6.85, 7.38 (m, 6H, 4H, stilbene protons); element analysis: Calc. for C_38_H_58_O_4_: C 78.90, H 10.03, O 11.07; found C 78.73, H 10.11, O 11.16%.

Compound **14M**: Yield: 34.7%; ^1^H-NMR (300 MHz, CDCl_3_, δ, ppm): 0.89 (t, 3H, –CH_2_–CH_3_), 1.29–1.81 (m, 35H, –CH_3_ on the oxetane ring, –OCH_2_(CH_2_)_6_CH_3_, –OCH_2_(CH_2_)_10_CH_2_–), 3.46 (t, 4H, –CH_2_OCH_2_–), 3.94 (t, 4H, –CH_2_–OPh–), 4.35, 4.50 (AB quartet, each 2H, –CH_2_– on the oxetane ring), 6.86, 7.39 (m, 6H, 4H, stilbene protons); element analysis: Calc. for C_39_H_60_O_4_: C 79.05, H 10.14, O 10.81; found C 78.92, H 10.20, O 10.88%.

### 2.3. Synthesis of Polymers **1P**~**14P**

In this study, all the polymers were synthesized by cationic ring-opening polymerization. The preparation of polymer is described below. Under nitrogen, dichloromethane was dried by calcium hydride and was distilled just prior to use. Boron trifluoride ether complex (freshly distilled) was used as an initiator. Under nitrogen, a solution of monomer (0.5 mmol) and dichloromethane (5 mL) was cooled to 0 °C and the initiator of 2% mol with respect to monomer was injected with a syringe. The reaction solution mixture was stirred at 0 °C for 24 h. Then, the resulting polymers were precipitated in methanol and purified further by dissolving in dichloromethane and then precipitating in ethanol repeatedly. The absence of monomer was checked by ^1^H-NMR and GPC.

## 3. Results and Discussion

This study intends to explore the liquid crystalline monomers of oxetane and liquid crystalline polyoxetane. Among the compounds of these monomers and polymers, spacer length and terminal chain length have influences on the thermal properties and mesophase. The molecular structure and the general synthetic procedures of the series of monomers are shown in Scheme 1, and all the products are examined by the nuclear magnetic resonance spectrometer and elemental analyzer in order to verify the correction of the molecular structure. There is one most important part among all the monomers in terms of using nuclear magnetic resonance spectrometer, that is oxetane in which the hydrogen, located on two carbons being beside the oxygen atoms, is the split from AB quartet, and it will gradually be disappearing after the ring-opening polymerization.

Monomers are composed of trans-stilbene as a mesogenic unit, their spacers length include several different alkyl chains (n = 3, 4, 5, 6, 12) and links with the terminals of different alkoxyl length (m = 1, 4, 5, 6, 7, 8). Moreover, there are fourteen monomers (**1M**~**14M**) in this series, and their structures are exhibited in Scheme 1. 

All monomers (**1M**~**14M**) used BF3. OEt2 as an initiator to carry out the ring-opening polymerization and successfully synthesized a series of brand-new side-chain liquid crystalline polymers (**1P**~**14P**), and their structures and their synthesis procedures are listed in Scheme 1.

The thermal and mesomorphic properties of monomers (**1M**~**14M**) and polymers (**1P**~**14P**) are measured using DSC, POM, and X-ray. The phase transition temperature and enthalpy changed of monomers **1M**~**14M** are reported in Table 1. The series of monomers (**1M**~**14M**) reveal enantiotropic smectic H and smectic G phases.

There is no obvious regularity in the change of the phase transition temperature of the monomer **1M**~**4M**, although the length of the spacer changes. This phenomenon may be affected by the volume of oxetane.

Table 1 illustrates the representative phase transition temperature of monomers **5M**~**9M** (containing six methylene units spacer length). As can be seen from Table 1 the tendency toward mesomorphic temperature range increase by increasing the length of alkoxy terminal group (**5M** is 2.6 °C, **6M** is 3.3 °C, **7M** is 2.1 °C, **8M** is 20.6 °C, **9M** is 38.1 °C). In addition, when the length of the terminal groups become shorter, the transition temperature of liquid crystalline will gradually overlap with the isotropic temperature. **5M**~**7M** are observed by POM, only the tiny transition of liquid crystalline phases can be seen. There is a tendency for the isotropic temperature to lower down the temperature from 136.4 to 118.0 °C when the length of terminal alkoxy is changed from m = 4 to m = 8. **10M**~**14M** (containing twelve methylene units spacer length) finds that the length of terminal alkoxy becomes larger, and the mesogenic temperature range becomes smaller (**10M** is 77.9 °C, **11M** is 56.9 °C, **12M** is 32.2 °C, **13M** is 22.6 °C, **14M** is 16.0 °C). This is the opposite result as compared with that of monomers **5M**~**9M**. This result may be due to the flexible spacer with a too larger length. From the above result, it is found that the mesomorphic temperature range has a great relationship with the spacer length. 

**Figure 1:**
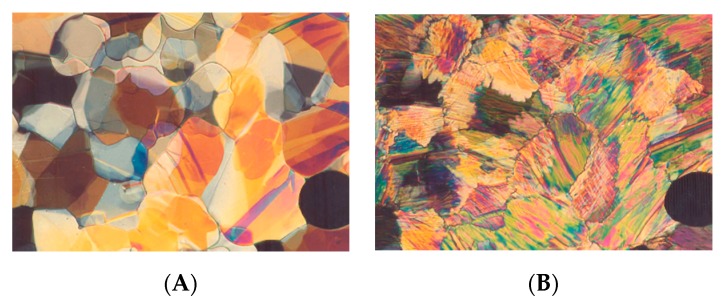
Optical polarizing micrographs displayed by monomer **9M** upon cooling from isotropic phase. (**A**) The smectic G mosaic platelets texture obtained at 115.6 °C (320×). (**B**) The smectic H zig-zag line texture obtained at 82.6 °C (320×).

**Figure 2:**
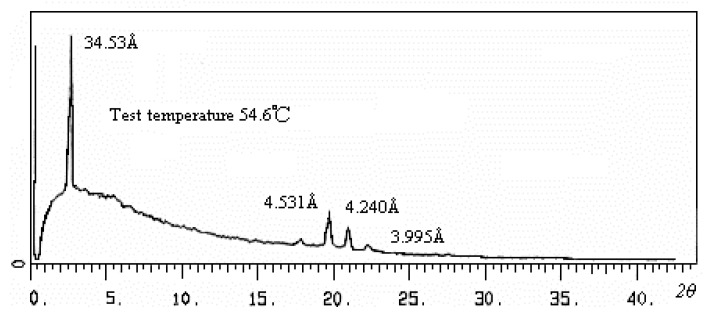
X-ray diffraction trace of **9M**.

Taking **9M** for an example, the dendritic growth pattern is developed at the temperature of 116.1 °C during the cooling scanning process, then mosaic platelets (Figure 1A) is formed at the temperature of 115.6 °C. At last, the liquid crystalline mesophase includes a Zig-Zag line shown at the diagram of mosaic platelets (Figure 1B) at the temperature of 82.6 °C. This characteristic is a transition phenomenon from smectic G phases to smectic H phases. In addition, in the X-ray test, Figure 2 presents the X-ray diffraction diagrams obtained from the powder sample of **9M** at 54.6 °C. The diffraction diagram presents a sharp first-order reflection at 34.53 Å, which is a layer of the length of smectic phase, and there are three diffraction peaks of 4.513, 4.240, and 3.995 Å in terms of the wider angles. Most of the X-ray diffraction studies performed on the SH phase indicate that it has a structure equivalent to that of the SE phase, except that the molecules have their long axes tilted concerning the normal to the layer planes. From the description of the phase as being of the SE type, it must be assumed that the molecules adopt an orthorhombic close-packing in a plane at right angles to the molecular long axes. Hence, because of the tilt, the pseudo-hexagonal net becomes even more distorted, and the phase has a monoclinic structure. According to the research of Volino, Dianoux and Hervet [17], the results of the X-ray test and the diagram represent smectic H phase.

**Figure 3:**
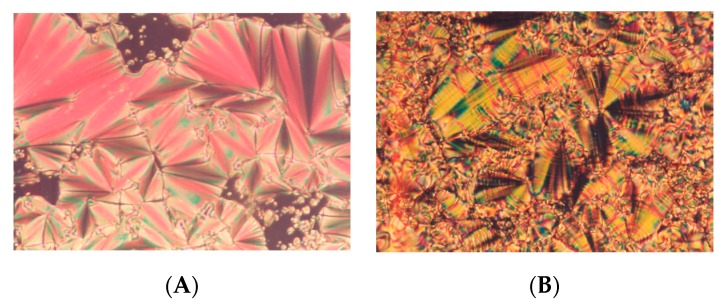
Optical polarizing micrographs displayed by polymer **14P** upon cooling from the isotropic phase. (**A**) The smectic A focal-conic texture obtained at 172.6 °C (320×). (**B**) The smectic E banded focal-conic texture obtained at 152.2 °C (320×).

Table 2 reports the thermal transition and thermodynamic parameters of polymers **1P**~**14P**. Polymers **1P**~**4P** and **6P**~**11P** exhibit enantiotropic smectic A phase, **5P** and **12P**~**14P** show enantiotropic smectic A and smectic E phase. Figure 3A,B exhibits the texture of polymer **14P** by optical polarizing micrograph. Figure 3A shows a focal conic fan texture of smectic A phase at 172.6 °C. Figure 3B displays the fissure focal cone fan shape of the texture of smectic E phase at 152.2 °C, while the temperature is cooling to 120 °C, the texture does not change except in the bigger crack. Therefore, it can be a regular liquid crystalline smectic texture of crystallization. Seen from Table 2, polymers (**1P**~**4P**) that contain a short flexible spacer (n = 3~6) and only one methyl terminal group show smectic A phase. Polymers (**5P**~**9P**, six methylene units spacer length; **10P**~**14P**, twelve methylene units spacer length) have different length of alkoxy terminal groups (m = 4~8). Their isotropic temperature and liquid crystalline transition temperature exhibits the same tendency. The isotropic temperature of **5P** and **10P** (m = 4) is especially high, maybe four alkoxy terminal groups can be easily arranged. **6P**~**9P** and **11P**~**14P** (m = 5~8) polymers increase in isotropic temperature with the length of the alkoxy terminal group. **11P**~**14P** more than **6P**~**10P** formed the regular liquid crystalline phase because they have a long flexible spacer.

The synthesized polymers (**1P**~**14P**) cannot be solved in an organic solvent (such as hexane, toluene, m-cresol, methanol, dichloromethane, acetone, ethyl acetate, THF, DMF, DMSO) under room temperature. During the ring-opening polymerization, the stilbene group might be cross-linked, because the total amount of H was decreased from the NMR spectrum after the polymerization, which might also cause the poor solubility of the synthesized polymer. In this study, **7P** and **14P** were selected for measuring molecular weight under the higher temperature of GPC, in which the solvent of m-cresol was used, and the test temperature of 85 °C was set. The molecular weight of **7P** is *M*_n_ = 4031, *M*_w_ = 5107, DPI = 1.266. The molecular weight of **14P** is *M*_n_ = 3666, *M*_w_ = 5052, DPI = 1.378. The results are presented as a ring-polymerization reaction by BF3. OEt2, the molecular weight of the obtained polymer is not high, but its dpi is small, as expected. The structure of the polymers is comb-like, and polystyrene was used as the standard of GPC, the test results from using GPC would be lower. However, in fact, the molecule weight should be higher.

## 4. Conclusions

In this study, a series of fourteen liquid crystalline monomers and polyoxetanes containing trans-biphenyl side group are successfully synthesized. It is known from the results that the liquid crystal phase transition temperature, the isotropic temperature, the stability of the liquid crystal phase, and the type of the liquid crystal all have a great influence on both the length of the soft spacer and the length of the terminal alkyl group. It is found that the difference in the length of the soft spacer and the length of the terminal alkyl group affect the monomer or polymer. When the length of the soft spacer and the length of the terminal alkyl group are increased, the liquid crystal phase formed is polymorphism of mesophases, such as **14M** shows *S_H_* and *S_G_*, **14P** shows *S_A_* and *S_E_*, and has a relatively stable liquid crystal phase.

## Figures and Tables

**Table 1:** Phase transition temperature and thermodynamic parameters of monomers **1M**~**14M**.

Compound	Phase Transitions, °C (Corresponding Enthalpy Changes, Kcal/mol) $\frac{heating}{cooling}$
**1M**	$\frac{S_{H}\;108.5{\left(-\right)}^{a}S_{G}117.1(4.63)\;I}{I\;111.6(-4.73)\;S_{G}102.0{\left(-\right)}^{a}S_{H}}$
**2M**	$\frac{S_{H}\;121.3{\left(-\right)}^{a}S_{G}124.5(3.92)\;I}{I\;120.9(-3.82)\;S_{G}116.0{\left(-\right)}^{a}S_{H}}$
**3M**	$\frac{S_{H}\;90.0{\left(-\right)}^{a}S_{G}118.0(3.68)\;I}{I\;113.0(-3.90)\;S_{G}81.6{\left(-\right)}^{a}S_{H}}$
**4M**	$\frac{S_{H}\;109.3{\left(-\right)}^{a}S_{G}114.3(3.97)\;I}{I\;104.9(-4.40)\;S_{G}101.6{\left(-\right)}^{a}S_{H}}$
**5M**	$\frac{S_{H}\;132.1{\left(-\right)}^{a}S_{G}136.4(6.94)\;I}{I\;132.6(-7.37)\;S_{G}130.0{\left(-\right)}^{a}S_{H}}$
**6M**	$\frac{S_{H}\;130.8{\left(-\right)}^{a}S_{G}132.2(8.05)\;I}{I\;130.6(-8.20)\;S_{G}127.3{\left(-\right)}^{a}S_{H}}$
**7M**	$\frac{S_{H}\;124.8{\left(-\right)}^{a}S_{G}126.2(4.77)\;I}{I\;123.9(-5.10)\;S_{G}121.8{\left(-\right)}^{a}S_{H}}$
**8M**	$\frac{S_{H}\;121.9{\left(-\right)}^{a}S_{G}122.8(9.52)\;I}{I\;117.6(-7.77)\;S_{G}97.7(-0.72)S_{H}}$
**9M**	$\frac{S_{H}\;97.0(0.72)S_{G}118.0(5.81)\;I}{I\;114.2(-6.37)\;S_{G}76.1(-0.29)S_{H}}$
**10M**	$\frac{S_{H}\;66.1(0.73)S_{G}131.6(7.19)\;I}{I\;126.7(-7.45)\;S_{G}48.8(-0.77)S_{H}}$
**11M**	$\frac{S_{H}\;64.0(1.29)S_{G}119.8(5.06)\;I}{I\;114.9(-5.14)\;S_{G}58.0(-1.02)S_{H}}$
**12M**	$\frac{S_{H}\;81.7{\left(-\right)}^{a}S_{G}116.6(8.08)\;I}{I\;110.0(-7.59)\;S_{G}77.8{\left(-\right)}^{a}S_{H}}$
**13M**	$\frac{S_{H}\;97.1{\left(-\right)}^{a}S_{G}121.2(8.86)\;I}{I\;115.8(-9.84)\;S_{G}93.2{\left(-\right)}^{a}S_{H}}$
**14M**	$\frac{S_{H}\;106.4{\left(-\right)}^{a}S_{G}123.5(14.0)\;I}{I\;119.1(-13.6)\;S_{G}103.1{\left(-\right)}^{a}S_{H}}$

*S_A_* = smectic A, *S_E_* = smectic E, *I* = isotropic. ^a^ determined by optical polarizing microscopic observation.

**Table 2:** Phase transition temperature and thermodynamic parameters of polymers **1P**~**18P**.

Compound	Phase Transitions, °C (Corresponding Enthalpy Changes, Kcal/mol) $\frac{heating}{cooling}$
**1P**	$\frac{K174.1{\left(-\right)}^{a}S_{A}\;178.0(4.08)\;I}{I\;173.6(-3.82)\;S_{A}170.2{\left(-\right)}^{a}K_{}}$
**2P**	$\frac{K185.1{\left(-\right)}^{a}S_{A}\;189.7(4.98)\;I}{I\;186.6(-4.90)\;S_{A}178.6{\left(-\right)}^{a}K_{}}$
**3P**	$\frac{K156.0{\left(-\right)}^{a}S_{A}\;152.2(4.52)\;I}{I\;146.6(-4.56)\;S_{A}135.4{\left(-\right)}^{a}K_{}}$
**4P**	$\frac{K121.5{\left(-\right)}^{a}S_{A}\;126.7(2.93)\;I}{I\;125.7(-2.61)\;S_{A}115.8{\left(-\right)}^{a}K_{}}$
**5P**	$\frac{K171.0{\left(-\right)}^{a}S_{E}188.5{\left(-\right)}^{a}S_{A}\;193.1(5.26)\;I}{I\;191.6(-4.83)\;S_{A}187.4{\left(-\right)}^{a}S_{E}168.5{\left(-\right)}^{a}K_{}}$
**6P**	$\frac{K166.4{\left(-\right)}^{a}S_{A}\;176.8(7.44)\;I}{I\;172.6(-6.99)\;S_{A}167.0{\left(-\right)}^{a}K_{}}$
**7P**	$\frac{K168.4{\left(-\right)}^{a}S_{A}\;179.5(8.53)\;I}{I\;174.6(-7.66)\;S_{A}170.1{\left(-\right)}^{a}K_{}}$
**8P**	$\frac{K168.7{\left(-\right)}^{a}S_{A}\;179.7(7.80)\;I}{I\;173.8(-7.60)\;S_{A}166.3{\left(-\right)}^{a}K_{}}$
**9P**	$\frac{K174.0{\left(-\right)}^{a}S_{A}\;183.9(7.27)\;I}{I\;176.4(-6.80)\;S_{A}167.3{\left(-\right)}^{a}K_{}}$
**10P**	$\frac{K169.8{\left(-\right)}^{a}S_{A}\;183.8(6.76)\;I}{I\;177.3(-7.11)\;S_{A}165.4{\left(-\right)}^{a}K_{}}$
**11P**	$\frac{K_{1}69.7(0.38)K_{2}90.4(0.27)K_{3}133.7{\left(-\right)}^{a}S_{A}\;138.6(6.78)\;I}{I\;130.4(-7.29)\;S_{A}122.4{\left(-\right)}^{a}K_{2}61.5{\left(-0.55\right)}^{}K_{1}}$
**12P**	$\frac{K_{1}84.6(0.46)K_{2}126.7(1.81)S_{E}162.1{\left(-\right)}^{a}S_{A}\;167.9(0.19)\;I}{I\;164.4(-0.53)\;S_{A}159.6{\left(-\right)}^{a}S_{E}123.1(-0.29)K_{2}61.6(-0.53)K_{1}}$
**13P**	$\frac{K_{1}93.9(1.42)K_{2}161.4{\left(-\right)}^{a}S_{E}167.3{\left(-\right)}^{a}S_{A}\;175.3(6.88)\;I}{I\;168.8(-7.19)\;S_{A}163.1{\left(-\right)}^{a}S_{E}158.5{\left(-\right)}^{a}K_{2}89.3(-0.88)K_{1}}$
**14P**	$\frac{K_{1}101.0(1.12)K_{2}164.5{\left(-\right)}^{a}S_{E}169.4{\left(-\right)}^{a}S_{A}\;177.4(7.08)\;I}{I\;171.6(-7.18)\;S_{A}164.8{\left(-\right)}^{a}S_{E}161.6{\left(-\right)}^{a}K_{2}98.2(-0.67)K_{1}}$

*S_A_* = smectic A, *S_E_* = smectic E, *I* = isotropic, *K* = crystal. ^a^ determined by optical polarizing microscopic observation.
